# Consequences on islet and incretin hormone responses to dinner by omission of lunch in healthy men

**DOI:** 10.1002/edm2.141

**Published:** 2020-04-28

**Authors:** Ola Lindgren, Bo Ahrén

**Affiliations:** ^1^ Department of Clinical Sciences Lund Lund University Lund Sweden

**Keywords:** C‐peptide, dinner, GIP, GLP‐1, glucagon, insulin, lunch

## Abstract

**Background:**

Omission of breakfast results in higher glucose and lower insulin and incretin hormone levels after both lunch and dinner. Whether omission of lunch has a similar impact on the following meal is not known.

**Aim:**

This study therefore explored whether omission of lunch ingestion affects glucose, islet and incretin hormones after dinner ingestion in healthy subjects.

**Materials & Methods:**

Twelve male volunteers (mean age 22 years, BMI 22.5 kg/m^2^) underwent two test days in random order with standard breakfast and dinner on both days with provision or omission of standard lunch in between.

**Results:**

The results showed that throughout the 300 minutes study period, glucose, insulin, glucagon and GIP levels after dinner ingestion did not differ between the two tests. In contrast, C‐peptide, and GLP‐1 levels were 26%‐35% higher at later time points after dinner ingestion when lunch had been omitted (*P* < .05).

**Conclusion:**

We conclude that omission of lunch increases GLP‐1 and insulin secretion and possibly also insulin clearance resulting in unchanged glucose and insulin levels after dinner ingestion.

## Graphical abstract

This study explored whether omission of lunch ingestion affects glucose, islet and incretin hormones after dinner ingestion in healthy subjects. Twelve male volunteers underwent two test days in random order with standard breakfast and dinner on both days with provision or omission of standard lunch in between. The results showed that omission of lunch increases GLP‐1 and insulin secretion and possibly also insulin clearance resulting in unchanged glucose and insulin levels after dinner ingestion.

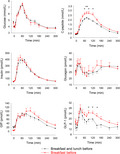

## 1 INTRODUCTION

It is known that timing of meal pattern is of importance for glycaemia throughout the day.^1^, ^2^, ^3^ In particular, timely ingestion of breakfast seems of particular important, since skipping breakfast has been demonstrated to result in enhanced postprandial glucose after lunch and dinner in association with a lower response in insulin and incretin hormones in subjects with type 2 diabetes.^4^ Similar data have been reported in healthy subjects.^5^ Furthermore, omission of breakfast increases 24 hours glycaemia in type 2 diabetes.^6^, ^7^, ^8^ These findings have been explained by the stimulation of insulin secretion by a first meal on a second meal,^8^, ^9^ coined as the ‘second meal effect’.^10^, ^11^ This beta‐cell memory has been suggested to be induced by previous glucose exposure.^11^

Also timing of ingestion of lunch has been shown to be of importance for glucose metabolism.^12^ Thus, ingestion of lunch late in the afternoon has been demonstrated to be associated with reduced insulin sensitivity and glucose tolerance in obesje and non‐obese humans compared with earlier intake of lunch.^13^, ^14^ However, there is no information on how metabolic responses to dinner ingestion are dependent on whether lunch is ingested or not. An approach to study this is to examine the consequence of omission of lunch ingestion on metabolic and hormonal effects of dinner ingestion.

We have undertaken a project in 12 healthy subjects who ingested standardized meals at different times. One report from this project was a study comparing metabolic and hormonal impact of ingestion of a standardized mixed meal at 8 am and 5 pm in healthy men showing that incretin hormone release has a diurnal pattern^15^ and another report showed that islet and incretin hormones are released by individual macronutrients in healthy men.^16^ In this project, it was also studied whether omission of lunch affected glucose and islet and incretin hormones after dinner. These data are reported here.

## 2 METHODS

### 2.1 Study population

Twelve lean male volunteers aged 20‐30 years (22.9 ± 1.9 (SD) years) and with a body mass index of 20‐25 kg/m^2^ (22.5 ± 1.5 kg/m^2^) were recruited through advertisement. They had no personal history of diabetes or gastrointestinal disease, and they were not taking any medication. The study was approved by the ethics committee of Lund University, Sweden (No 607/2005), and all subjects gave written informed consent before entry into the study.

### 2.2 Study protocol

The study subjects attended the research department twice after an overnight fast with no food after 10 pm in random order for the two tests. At both occasions, subjects attended the clinical research unit at 8 am and stayed until the evening. At both occasions, the subjects received a breakfast at 8 am. The breakfast was standardized and consisted of 524 kcal (19% protein, 18% fat, 63% carbohydrates) as rye and wheat bread wholemeal (fibre content 67%) (60 g), margarine (fat 40%; 10 g), smoked ham from pork (fat 3%, 15 g), cheese (fat 17%, 15 g), orange juice (150 g), green pepper (40 g), light sour milk (fat 0.5%; 200 g) and muesli with fruit (40 g). Then, subjects received in random order either a standard lunch at 1 pm or no lunch was provided. The lunch consisted of 606 kcal (25% protein, 38% fat, 37% carbohydrates) as filet of cod (Alaska pollock, 124 g), mashed potatoes (197 g) and light sour milk (fat 0.5%, 200 g). In the afternoon, subjects were again provided with an antecubital vein catheter. After two baseline samples were collected, a standard dinner was rapidly ingested at 5 pm. Additional blood samples were taken throughout a 300‐minute study period at times 5, 10, 20, 30, 45, 60, 75, 90, 105, 120, 150, 180, 240 and 300 minutes after meal ingestion. The standard dinner consisted of 690 kcal (25% protein, 35% fat, 40% carbohydrates) as potatoes (150 g), brown sauce made of powder, milk, water (70 g), lingonberryjam (60 g), green beans (70 g), light milk (fat 0.5%, 200 g), apple with peel (125 g), porkcutlet (fatty rim 5 mm, 125 g), hard bread (rye, fibre content 15.5%) (12 g) and margarine (fat 40%, 10 g). Two weeks later, the subjects returned and were given the standard breakfast followed by lunch or no lunch and then the dinner with similar blood sampling, so that ingestion or omission of lunch was provided in a cross‐over design. There was no restriction on physical activity the day before the test, but during the test day, no physical exercise or activity was allowed, although the subjects were permitted to sit in between the meal tests. Also, the subjects were not allowed to have any snacks in between meals during the test days. The power analysis was based on the assumption to have an 80% probability of detecting a difference of 30% between glucose and insulin levels after meal ingestion with a *P* < .05. With the narrow group of having healthy young men and using a cross‐over design where each individual subjects is his own control, the power analyses stated at least 10 subjects are required. We added two extra and recruited therefore twelve subjects for the study.

### 2.3 Analyses

Blood samples, collected in chilled tubes containing EDTA (7.4 mmol/L) and aprotinin (500 kIU/mL; Novo Nordisk), were immediately centrifuged at 4°C and plasma was frozen at −20°C. Glucose was measured using the glucose oxidase method. Insulin, C‐peptide and glucagon were analysed with double‐antibody RIA (Linco Research). Blood samples for determining GIP (glucose‐dependent insulinotropic peptide) and GLP‐1 (glucagon‐like peptide‐1) were collected into chilled tubes containing EDTA and aprotinin with addition of diprotin A (0.1 mmol/L; Bachem) and determined with ELISA (Merck Millipore). The assays of GIP and GLP‐1 are based on antibodies directed to different parts of the molecules and cross‐react to 100% with GIP (1‐42) and GIP (3‐42), and GLP‐1 (7‐36) and GLP‐1 (9‐36), respectively, and therefore reflect the total values of the incretin hormones. The GIP assay does not significantly cross‐react with glucagon, oxyntomodulin, GLP‐1 or GLP‐2. The GLP‐1 assay has no significant cross‐reactivity with GIP, GLP‐2, glucagon or oxyntomodulin.

### 2.4 Estimations and statistics

Means ± SEM are shown. Suprabasal (incremental) areas under curves (AUC) were calculated by the trapezoid rule for suprabasal levels during 0‐30 minutes (early response), during 30‐300 minutes (late response) and during the entire 300 minutes (total response) after meal ingestion. OGIS (oral glucose insulin sensitivity index) was estimated as a surrogate for insulin sensitivity.^17^ Glucose‐stimulated insulin secretion (GSIS; 30 minutes increase in C‐peptide levels divided by 30 minutes increase in glucose levels)^18^ and adaptation index (OGIS times GSIS)^19^ were also determined. Insulin clearance was assessed by using the molar ratio of C‐peptide to insulin^20^, ^21^ and estimated as 1 minus the ratio of AUC_insulin_ to AUC_C‐peptide_ times 100. One‐way ANOVA was used for testing significance of the time curves, and Student's paired two‐tailed test was used for tests of significance between other parameters.

## 3 RESULTS

### 3.1 Glucose, insulin, C‐peptide and glucagon

**FIGURE 1:**
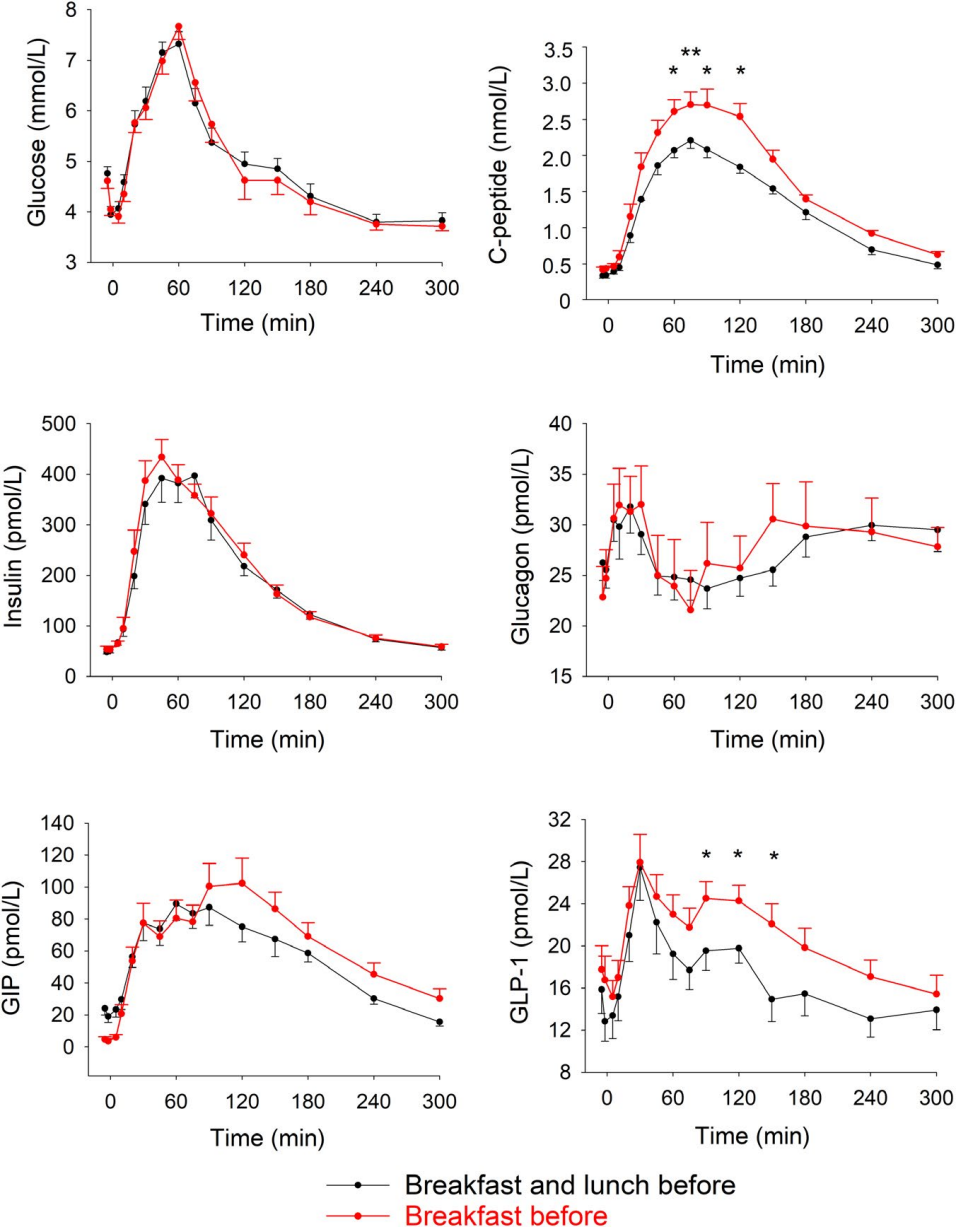
Plasma levels of glucose, insulin, C‐peptide, glucagon, GIP and GLP‐1 before and during 300 min after dinner ingestion when breakfast and lunch or only breakfast had been ingested before the dinner on the same day in 12 healthy male volunteers. Means ± SEM are shown. Asterisks show the probability level of random difference between the two tests as obtained by paired Student's *t* test. *P < .05, **P < .01

Glucose levels before and after dinner ingestion were not different between the two test days, that is, not dependent on whether or not lunch had been ingested. Similarly, insulin levels before and after dinner ingestion were not different between the two days. Furthermore, C‐peptide levels did not differ between the two tests during the initial 60 minutes after meal ingestion. In contrast, plasma C‐peptide levels were significantly higher at 60‐120 minutes after dinner ingestion when lunch had been omitted. Glucagon levels did not differ between the two test days. AUC_glucose_, AUC_insulin_, AUC_C‐peptide_ and AUC_glucagon_ did not differ significantly between the test days neither when calculated for the 0‐30 minutes, 30‐300 minutes or total 300 minutes period (Figure 1, Table 1).

**Table 1:** Suprabasal (incremental) area under the curves (AUC) for levels of glucose, insulin, C‐peptide, glucagon, GIP and GLP‐1 and estimated insulin clearance during 0‐30 min (early), 30‐300 min (late) and the entire 0‐300 min period (total) and OGIS, GSIS and adaptation index after dinner ingestion when breakfast and lunch or only breakfast had been ingested before the dinner on the same day

	Breakfast and lunch	Breakfast only, no lunch	*P*
AUC_glucose_ (mmol/L min)			
Total	243 ± 35	215 ± 43	.52
Early	37.8 ± 4.9	30.2 ± 2.9	.31
Late	204 ± 35	185 ± 42	.62
AUC_insulin_ (nmol/L min)			
Total	38.1 ± 3.3	38.9 ± 2.6	.64
Early	4.2 ± 0.6	4.7 ± 0.7	.19
Late	33.9 ± 3.0	34.1 ± 2.3	.76
AUC_C‐peptide_ (nmol/L min)			
Total	251 ± 13	317 ± 21	.27
Early	14.6 ± 1.6	19.0 ± 3.0	.092
Late	236 ± 13	298 ± 20	.039
AUC_glucagon_ (pmol/L min)			
Total	805 ± 317	824 ± 425	.25
Early	175 ± 50	190 ± 46	.55
Late	650 ± 286	615 ± 433	.85
AUC_GIP_ (nmol/L min)			
Total	11.4 ± 1.8	13.7 ± 2.3	.27
Early	0.86 ± 0.15	1.13 ± 0.19	.13
Late	10.6 ± 1.8	13.4 ± 2.4	.32
AUC_GLP‐1_ (nmol/L min)			
Total	0.82 ± 0.36	1.01 ± 0.43	.88
Early	0.18 ± 0.042	0.14 ± 0.039	.48
Late	0.64 ± 0.32	0.87 ± 0.41	.31
Insulin clearance (% extraction)			
Total	85.1 ± 1.1	87.5 ± 0.9	.11
Early	72.2 ± 0.9	74.1 ± 0.8	.18
Late	85.4 ± 0.9	90.2 ± 0.7	.04
OGIS (mL/min/m^2^)	477 ± 19	486 ± 9	.65
GSIS (nmol/mmol)	0.82 ± 0.24	1.03 ± 0.18	.38
Adaptation index (OGIS × GSIS)	384 ± 115	511 ± 98	.23

Note

OGIS (a surrogate for insulin sensitivity), glucose‐stimulated insulin secretion (GSIS; 30 min increase in C‐peptide divided by the 30 min increase in glucose) and adaptation index (OGIS times GSIS) after dinner ingestion when breakfast and lunch or only breakfast had been ingested before the dinner on the same day. The study was undertaken in 12 healthy male volunteers. Means ± SEM are shown. *P* shows the probability level of random difference between the two tests as obtained by paired Student's *t* test.

### 3.2 Insulin clearance, insulin sensitivity and insulin secretion

Insulin clearance was assessed as 1 minus the ratio of AUC_insulin_ to AUC_C‐peptide_. This surrogate measure was numerically higher when lunch had been omitted compared to when lunch had been ingested, both when using the early AUCs, the late AUCs and the total AUCs, although the difference was significant only for late AUCs. Estimated insulin sensitivity (OGIS), GSIS and adaptation index (relating beta‐cell function to insulin sensitivity) did not differ significantly between the two tests (Table 1).

### 3.3 GIP and GLP‐1

GIP levels did not differ significantly between the two tests, and GLP‐1 levels were not different during the initial 90 minutes after meal ingestion. However, GLP‐1 levels after dinner were significantly higher at 90‐150 minutes when lunch had been omitted. AUC_GIP_ and AUC_GLP‐1_ did not differ significantly between the test days (Figure 1, Table 1).

## 4 DISCUSSION

The main finding in this study is that glucose and insulin levels after dinner are the same regardless of whether lunch has been ingested or not. This shows that omission of lunch is not disrupting the metabolism such that dinner responses in glucose or insulin are affected. This is therefore different from the impact of breakfast, as evident by earlier studies showing higher glucose and lower insulin levels following lunch and dinner after omission of breakfast.^4^, ^5^, ^6^ This suggests that breakfast ingestion has a greater impact on glucose and insulin homeostasis than lunch ingestion.

Some differences were observed, however, when comparing responses to dinner ingestion with or without a preceding lunch ingestion. One interesting, although seemingly paradoxical, finding was that C‐peptide levels were enhanced after dinner by omission of lunch yet insulin levels were not affected. This would suggest that insulin secretion is increased (as reflected by the higher C‐peptide), and at the same time, insulin clearance is also enhanced (as reflected by failure of insulin to be increased when C‐peptide levels are increased). We therefore estimated these processes. To estimate insulin secretion, we used glucose‐stimulated insulin secretion (GSIS) by analysing the 30‐minute increase in C‐peptide levels divided by the 30 minutes increase in glucose levels.^18^ There was no significant difference between the two tests in GSIS suggesting that insulin secretion is not dependent on whether lunch has been consumed or not. This is also supported by our estimation of the adaptation index. It is well known that beta‐cell secretion is dependent on insulin sensitivity such that in insulin resistance insulin secretion is increased.^22^ An accurate determination of insulin secretion as surrogate for beta‐cell function therefore requires normalization for insulin sensitivity. This may be performed by multiplying insulin levels times insulin sensitivity, which is the basis for the disposition index.^23^ However, since this index is based on peripheral insulin levels, it includes both secretion and clearance of insulin. When instead C‐peptide levels have been measured, as in this study, it is preferable to use the adaptation index, which is an index relating insulin secretion to insulin sensitivity, without the complication of involving also insulin clearance.^19^ To do this, we first estimated insulin sensitivity during dinner ingestion and we used an index based on the dynamic changes of glucose and insulin during the meal, the OGIS. The OGIS index has been shown to be preferable to other indices.^24^ OGIS was initially developed for estimation of insulin sensitivity after oral glucose^17^ but has also been used after meal ingestion.^25^ We found that OGIS was not significantly different between the two tests, and when we multiplied OGIS by GSIS for estimation of adaptation index, we also found that this index was not significantly different. Therefore, we conclude that insulin secretion and beta‐cell function are not altered after dinner whether lunch has been ingested or not.

The finding that insulin secretion was not different between the two tests yet C‐peptide levels were enhanced but insulin levels were the same suggests that insulin clearance after dinner is enhanced when lunch is omitted. This was supported by the significantly higher value of the surrogate for insulin clearance when lunch had been omitted when using the 30‐300 minutes time interval. Insulin clearance is mainly executed in the liver,^26^ and our results therefore suggest a higher hepatic insulin extraction after dinner after omission of lunch. Previous studies have shown that GIP reduces but GLP‐1 increases insulin extraction in humans.^27^ One possibility would be that the lower GIP levels that must have occurred during afternoon hours when lunch is omitted, compared to when lunch is ingested, could have induced a higher hepatic extraction which would persist throughout the dinner period. Alternatively, the higher GLP‐1 secretion after dinner would contribute to an increased insulin clearance. However, there is no substantial evidence for these two hypothesises, and therefore, more specific studies are required to further explore this finding. It may also be speculated that the increased insulin clearance in the context of increased insulin secretion is a mean to avoid hyperinsulinaemia after the dinner, since hyperinsulinaemia would have potentially contributed to insulin resistance.

Another finding in this study was that GLP‐1 levels after dinner ingestion were slightly elevated when lunch was omitted. These higher values were observed at 90‐150 minutes after dinner ingestion. The result raises the question how GLP‐1 secretion is regulated and which of the factors involved in this regulation that may be perturbed by omission of lunch. A main mechanism for GLP‐1 secretion is meal size and composition. Thus, a large meal results in higher GLP‐1 secretion than a smaller meal with the same composition.^28^ Furthermore, incretin hormone secretion seems particularly sensitive to carbohydrate ingestion.^29^, ^30^ Also rate of gastric emptying seems important, since a more rapid emptying results in a more rapid nutrient presence in proximity to enteroendocrine cells resulting in enhanced secretion.^31^, ^32^ On the other hand, more rapid meal ingestion does not seem to affect GLP‐1 secretion.^33^ Meal timing is also important, as evident from a study showing that incretin hormone secretion after similar meals is higher in the morning than in the afternoon.^15^ Physical activity may be important as evident by a study showing that the GLP‐1 response to oral glucose is enhanced by physical activity^34^ and also prolonged fasting may also be important, since skipping breakfast has been shown to result in a diminished GLP‐1 response to lunch.^4^ In addition, disease states may be important.^30^ In the present study, all subjects were healthy, the ingested dinners had exactly the same composition in the two tests and the dinners were ingested at the same time of the day. Physical activity was also not different between the days. Remaining difference which may impact incretin hormone secretion is the extended fasting which occurs when lunch is omitted and perturbations induced by the ingested lunch which are not induced. Since extended fasting would be expected to diminish incretin hormone secretion,^4^ it is not likely the explanation for the higher incretin hormone response to dinner when lunch is omitted. Instead, responses to lunch could be of relevance. One such consequence could be that GLP‐1 levels are higher during the hours after lunch ingestion compared to the day when lunch is omitted. This could have affected the gastric emptying in that the higher GLP‐1 levels would have diminished gastric emptying. When GLP‐1 levels are lower during the hours when lunch was omitted, a more rapid gastric emptying after dinner would be a possibility, which would result in higher incretin hormone levels. This hypothesis can be tested in further studies, as would other potential differences.

It may seem paradoxical that insulin levels after dinner were not enhanced when lunch had been omitted considering that incretin hormones levels were enhanced, since GLP‐1 is known to stimulate insulin secretion by both a direct effect on beta cells and through the vagus nerves.^35^ There are, however, several potential explanations for this. One is that glucose levels were declining at the time points when GLP‐1 levels were enhanced, which might have counteracted an insulinotropic action of GLP‐1. Another explanation would be that the difference in GLP‐1 levels between the two tests was small and below an efficient level. Also, we determined total levels of GLP‐1, which not necessarily reflects the active levels, which would have required a measurement of the intact form.^36^ It may also be argued that the enhanced GLP‐1 levels indeed contributed to the increased C‐peptide levels but that the failure to enhance also insulin levels depends on increased insulin clearance.

The strength of this study is the cross‐over design which allows that each participant serves as his own control. Another strength is the standardization of not only the dinner content, but also of the breakfast content. A limitation of the study is its short‐term nature, due to which it is not possible to conclude whether a more persistent omission of lunch also would impact metabolic responses to dinner ingestion. Another limitation is that the study was undertaken only in healthy, young men, and therefore, the generalization also to women, to older subjects and to subjects with diseases such as obesity and or type 2 diabetes cannot be performed. Furthermore, a third limitation might be that the standardized dinner was of exactly the same size and composition in all subjects, and therefore, the meal ingestion was not related to individual daily energy needs. However, the standardization of the meal could also be an advantage, since the responses to the dinner reflect the responses to the same nutritional challenge. In addition, the study might have been underpowered for some of the measures, such as GLP‐1 concentrations, which were different between the tests at some time points but not when examining the entire AUC. Finally, we did not undertake an appetite assessment during the two test days, and therefore, it is not possible to conclude how the subjects appetite changed by omission of lunch.

In conclusion, omission of lunch increases GLP‐1 and insulin secretion and possibly also insulin clearance resulting in unchanged glucose and insulin levels after dinner ingestion.

## CONFLICT OF INTEREST

The authors have nothing to declare.

## AUTHOR CONTRIBUTIONS

The study was designed by BA and conducted by OL and BA who also collected and analysed data and wrote the manuscript. BA is the guarantor of the study.

## Data Availability

The data that support the findings of this study are available from the corresponding author (BA), upon reasonable request.
